# Supplementary material for: Improved Efficiency of Perovskite Solar Cells by the Interfacial Modification of the Active Layer
Source: Nanomaterials (Basel). 2019 Feb 5;9(2):204. doi: 10.3390/nano9020204 (PMC6410319; doi:10.3390/nano9020204)
Supplement: Supplementary file 1 [file nanomaterials-09-00204-s001.pdf]

nanomaterials

Supplementary Material:

Improved Efficiency of Perovskite Solar Cells by the Interfacial Modification of the Active Layer

Zhen Lu[1], Shangzhi Wang[1], Huijun Liu[1], Feng Feng[1,*] and Wenhua Li[2,*]

1 College of Chemistry and Environmental Engineering, ShanXi DaTong University, Datong 037009, China;
2 Beijing Key Laboratory of Energy Conversion and Storage Materials, College of Chemistry, Beijing Normal University, Beijing 100875, China

* Correspondence: feng-feng64@263.net (F. F.); 11112013119@bnu.edu.cn (W. L.)

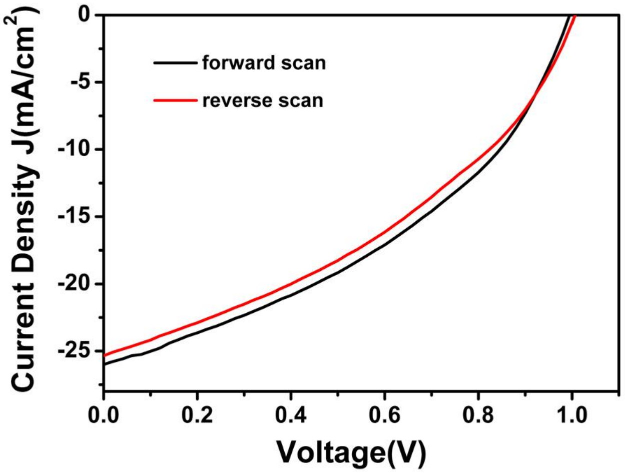

Fig S1. J-V curves of PSCs with DMSO-treated PEDOT:PSS layer (PVSK/DMSO/PVSK). The devices were scanned along the forward and reverse directions to study the hysteresis, with a delay time of 20 ms and a voltage step of 0.02 V s^{-1}.

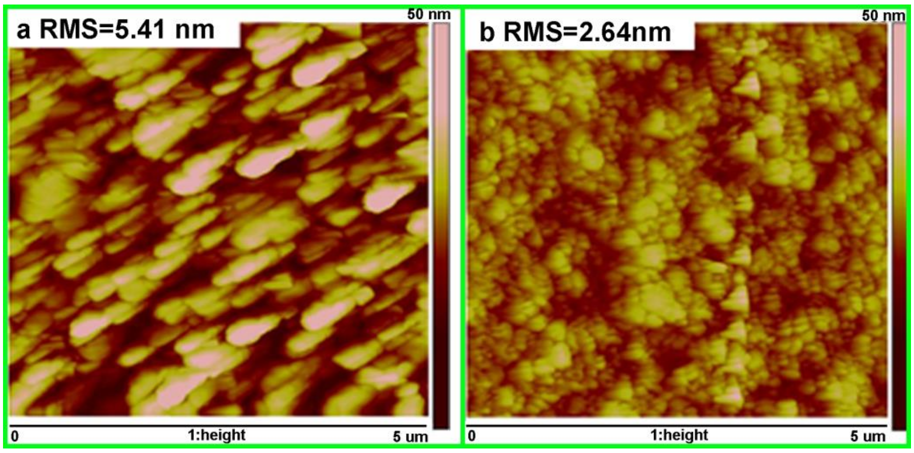

Fig S2. AFM image of (**a**) perovskite film processed on a pristine PEDOT:PSS film; (**b**) perovskite film processed on PEDOT:PSS film coated with the perovskite layer washing away by DMSO.

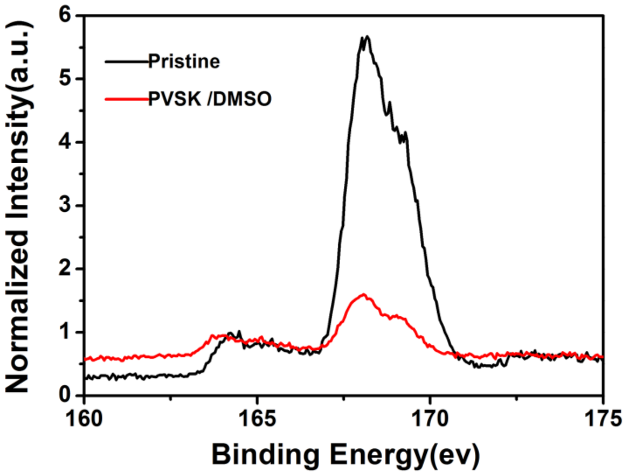

Fig S3. 2p XPS spectra of a pristine PEDOT:PSS film and PEDOT:PSS films treated with perovskite layer rinsed away with DMSO.

Table S1. Performance data of PSCs on a perovskite layer rinsed away with DMSO(PVSK/DMSO/PVSK) at the optimal conditions (annealing temperature : 50 ^{o}C, time : 5 min).

Samples	V_{oc}(V)	J_{sc}(mA cm^{-2})	FF	PCE(%)
1	0.950	21.6	0.519	10.68
2	0.950	20.65	0.520	10.25
3	0.957	21.77	0.537	11.20
4	0.960	21.90	0.539	11.36
5	0.958	21.80	0.509	10.66
6	0.961	22.00	0.501	10.66
7	0.950	21.75	0.538	11.13
8	0.960	20.62	0.525	10.60
9	0.965	21.40	0.535	11.05
10	0.959	22.32	0.514	11.03
11	0.977	22.50	0.514	11.31
12	0.955	20.65	0.530	10.50
13	0.950	20.50	0.525	10.24
14	0.960	21.70	0.537	11.21
15	0.970	21.30	0.530	11.03
16	0.960	21.50	0.539	11.15
17	0.975	21.40	0.531	11.05
18	0.975	21.70	0.534	11.32
19	0.970	21.50	0.539	11.28
20	0.975	21.40	0.538	11.25

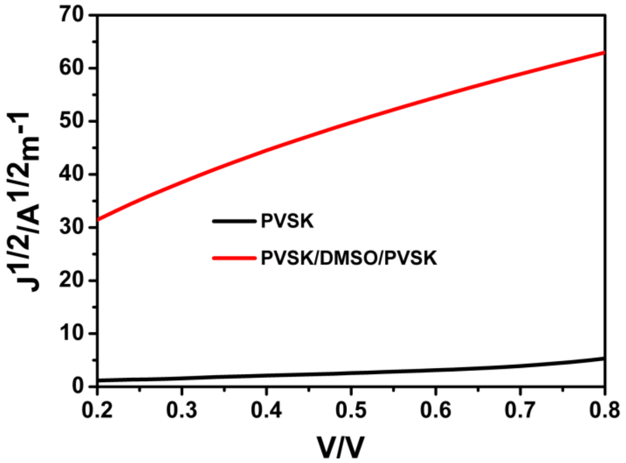

Fig S4. SCLC curves of devicess on a pristine PEDOT:PSS film (PVSK) and PEDOT:PSS films treated with perovskite layer rinsed away with DMSO (PVSK/DMSO/PVSK).

© 2019 by the authors. Submitted for possible open access publication under the terms and conditions of the Creative Commons Attribution (CC BY) license (http://creativecommons.org/licenses/by/4.0/).
